# Standards for permanent tooth emergence in Czech children

**DOI:** 10.1186/s12903-017-0427-9

**Published:** 2017-11-29

**Authors:** Romana Šindelářová, Lucie Žáková, Zdeněk Broukal

**Affiliations:** Institute of Dental Medicine, 1st Medical Faculty of Charles University, Prague, Czech Republic

**Keywords:** Permanent teeth, Emergence time, Czech Republic, Children

## Abstract

**Background:**

The aim of this study is to develop a population-specific reference for permanent tooth emergence among 4–15 years old Czech Republic children. The reference derived from this sample population are essential for pediatric dental diagnostics, orthodontic treatment planning, as well as anthropological and forensic applications.

**Methods:**

In this cross-sectional epidemiological survey, dental examinations of 1,370 Czech children (696 girls (50,8%) and 674 boys (49,2%), whose parents or legal guardians all signed informed consent forms) from 11 elementary schools and kindergartens were performed in the classroom. During the examination, previously emerged permanent teeth (other than third molars), the child's age and gender were recorded. A tooth is defined as having erupted when at least any part of the crown penetrates the gingiva, and is clinically seen in the oral cavity. A logistic regression model was used to calculate the median emergence age per tooth for both genders and the total sample. The data was statistically processed (using IBM SPSS Statistics 23) and carried out at a deviation level of 0.05. The statistical significance of the differences in the emergence of permanent teeth (contralateral, intermaxillary, inter-gender differences) was evaluated by the McNemar test.

**Results:**

No statistically significant differences were observed in the emergence times between right and left sides. In addition, mandibular teeth emerged earlier than the corresponding maxillary ones (with the exception of the first and second premolars). Permanent teeth were found to emerge sooner in girls. Furthermore, the greatest inter-gender difference was observed in maxillary canines.

**Conclusions:**

The new data reported can now be used as standards when assessing permanent tooth emergence of Czech children.

## Background

The emergence times and sequences of eruption of permanent teeth are important in assessing child growth and development. It is very useful for dental treatment planning, particularly in orthodontics, pediatric, physiologic age estimation and human identification in forensic dentistry. Tooth eruption occurs when the forming tooth migrates from its intraosseous location in the jaw to its functional position within the oral cavity [1]. The first set of teeth to erupt is called the primary dentition (deciduous teeth). This is followed by a period of mixed dentition which begins with the eruption of the first permanent tooth, and ends when the last deciduous tooth is replaced. The replacement of deciduous teeth occurs in two stages. Eruption phase I is characterized by the eruption of the first permanent molars and incisors, while during eruption phase II, canines, premolars and second permanent molars emerge. An understanding of the sequence of tooth emergence in phase II is important for the interceptive extraction of the first premolar in cases of crowding. The preferred order is first premolar, canine and then second premolar, which is not usual in the mandible [2–4]. Additionally, in some populations [2], maxillary second premolars could emerge before canines which could be the cause for the lack of space for canines. Another change in the order could be the emergence of second molars before the second premolars, this array could push mesially on the first molar and cause the lack of space for the eruption of second premolars in which case orthodontic treatment would be necessary. The timing and sequence of permanent tooth emergence are influenced by gender, racial, genetic and environmental factors. Most studies reported female advancement and this has been credited to earlier onset of development in females. Furthermore, studies have shown earlier eruption times in sub-Saharan African population compared to Asian [5] and Caucasian population [6]. This has been attributed mainly to genetic differences. Environmental influences such as smoking during pregnancy [7], climate [8], circadian rhythms [9], fluoride intake [10, 11], premature loss of deciduous teeth [12–14] systemic disease [15, 16], seasons of the year [17], nutrition [18, 19], physical constitution, morphology of the craniofacial region, premature birth [20] and socioeconomic factors [21, 22] have also been implicated as a source of variations in timing of permanent tooth emergence.

This is the first study to provide population specific standards for timing and sequence of emergence of permanent teeth in the Czech Republic. To date dental practitioners from Czech Republic, rely on references derived from other populations which may not be directly applicable to Czech Republic populations due to environmental and genetic differences.

## Methods

### Study population

This cross-sectional study was conducted over the years 2013 and 2014 in 11 elementary schools and kindergartens throughout the Czech Republic. The sites were pre-selected in accordance with the WHO guidelines for national cross-sectional oral-health surveys, so that representative sample of urban and rural school children aged 4–15 years is included in the study sample. The study was approved by the Ethics Committee of the General University Hospital in Prague and the data collected was in accordance with Act No. 101/2000 Coll. on the Protection of Personal Data. Permission to carry out the study was obtained from the respective school heads. Informed Consent was obtained from parents or legal representatives while assent was obtained from the children. A total number of 1370 children whose parents are natives of Czech Republic and other minorities of Caucasian origin (Moravian, Silesian, Slovak, Polish, German, Hungarian) [23, 24] who live in the Czech Republic were included in the study. Children of Mongoloid race (mostly Vietnamese minority) and Negroid race were excluded.

### Data collection

Methodological procedures for data collection were selected according to the protocols of similar studies conducted in other countries [25, 2]. All the selected participants were given a two-minute epidemiological dental examination directly in their classrooms. All erupted teeth (other than third molars), age (with an accuracy to two decimal points) and gender were recorded. Each emerged permanent tooth in the oral cavity was recorded and identified according to the FDI [26] two-digit system and was classified into two grades: Grade 0 - tooth is not visible within the oral cavity and Grade 1 - clinically visible permanent tooth within the oral cavity.

### Statistical data analysis

**Fig. 1:**
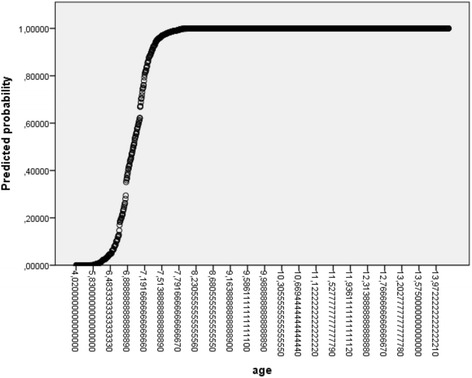
Probability of occurrence of tooth 11 according to the chronological age (logistic regression model)

Several statistical methods, probit analysis, Kärbers’ method, life tables and maximum likelihood analysis, are all appropriate to determine the mean age and range at which 50% of the children are most likely to have a tooth emerged (equivalent of the 50th percentile) [27]. In this study, binary logistic regression models (28 models for all children, 28 for boys and 28 for girls) were applied for the computation of median age of emergence per tooth. The statistical analysis was performed using statistical software (IBM SPSS Statistics 23). All statistical tests were performed at the 0.05 level of significance. The 0.05 and 0.95 probability of response level were displayed reflecting the 5th and 95th percentiles. The independent variable was the chronological age of subjects and the dependent variable was the dichotomous response the output of the erupted/unerupted tooth. The equation for logistic regression: ln(P/(1-P)) = b_0_ + b_1_xAGE, where P is the probability of the emergence of the individual tooth, b_0_ is the constant and b_1_ is the regression coefficient for variable age. As an example, the estimates and the chart of probabilities (Fig. 1) for tooth 11 are presented below. After the estimation of b_0_ and b_1_ for the median of all 3 × 28 models, P5 and P95 were computed according to the following formulas: P_5_ = −b_0_/b_1_; P_5_ = (ln(0,05/0,95)-b_0_)/b_1_; P_95_ = (ln(0,95/0,05)-b_0_)/b_1_. The statistical significance of the differences in the emergence pattern of teeth (contralateral, intermaxillary and inter-gender) was evaluated by the McNemar test. Previous studies mostly applied the Mann-Whitney U test [28], but this strategy is not fully appropriate because this test can only be applied in two independent samples and only compares median values. The contingency tables of presence/absence of each tooth were made for the whole data set. The McNemar test criteria follows Chi/square distribution with one degree of freedom (the critical value for significance 0,05 is therefore 3,84) and compares the number of children in which only tooth 11 has emerged with those in which only tooth 21 has emerged. If these numbers are comparable, the McNemar test results are statistically insignificant and vice versa.

## Results

Table 1 gives the median ages, 5th and 95th percentiles for emergence of permanent teeth. The data is provided for the total sample and for each gender separately.

**Table 1:** The standards of permanent teeth emergence (for right and left sides separately) for the total sample, girls and boys and the inter-gender differences

Tooth			Time of emergence									Odd	Sig	
			Total			Boys			Girls					
			Median	Percentile		Median	Percentile		Median	Percentile				
				5th	95th		5th	95th		5th	95th			
Right side	Maxillary	Central incisor	6.97	6.49	7.44	7.04	6.72	7.36	6.89	6.32	7.46	2.389	.017	Sig
		Lateral incisor	7.82	6.57	9.07	8.03	6.73	9.33	7.61	6.53	8.70	2.709	.000	Sig
		Canine	10.88	9.38	12.38	11.32	9.98	12.67	10.45	9.19	11.71	7.234	.000	Sig
		First premolar	9.46	8.29	10.62	9.55	8.40	10.69	9.37	8.22	10.52	1.570	.085	NS
		Second premolar	10.92	9.54	12.30	10.98	9.59	12.38	10.86	9.51	12.21	1.293	.276	NS
		First molar	6.74	6.15	7.33	6.86	6.35	7.37	6.62	5.98	7.26	3.117	.001	Sig
		Second molar	12.57	11.13	14.02	12.67	11.03	14.32	12.48	11.25	13.71	1.402	.160	NS
	Mandibular	Central incisor	6.32	5.43	7.22	6.41	5.57	7.25	6.21	5.24	7.18	1.684	.124	NS
		Lateral incisor	7.25	6.52	7.99	7.34	6.60	8.07	7.18	6.47	7.88	1.930	.021	Sig
		Canine	9.26	8.16	10.35	9.41	8.33	10.49	9.12	8.06	10.17	2.283	.003	Sig
		First premolar	9.88	8.75	11.00	10.01	9.00	11.02	9.75	8.56	10.93	1.993	.009	Sig
		Second premolar	10.75	9.42	12.09	10.89	9.49	12.29	10.62	9.40	11.84	1.822	.014	Sig
		First molar	6.34	5.47	7.21	6.47	5.69	7.25	6.17	5.19	7.14	2.254	.019	Sig
		Second molar	12.11	10.34	13.88	12.41	10.66	14.17	11.81	10.16	13.45	2.830	.000	Sig
Left side	Maxillary	Central incisor	6.98	6.56	7.40	7.02	6.88	7.16	6.93	6.37	7.49	1.834	.108	NS
		Lateral incisor	7.81	6.54	9.08	8.04	6.75	9.34	7.58	6.49	8.67	3.001	.000	Sig
		Canine	10.92	9.42	12.41	11.38	10.06	12.69	10.47	9.23	11.72	7.965	.000	Sig
		First premolar	9.46	8.31	10.62	9.56	8.49	10.64	9.37	8.17	10.57	1.632	.063	NS
		Second premolar	10.88	9.49	12.27	10.98	9.47	12.50	10.78	9.55	12.02	1.517	.078	NS
		First molar	6.73	6.08	7.38	6.87	6.33	7.41	6.58	5.85	7.30	3.443	.000	Sig
		Second molar	12.68	11.01	14.36	12.77	11.22	14.31	12.60	10.83	14.37	1.393	.144	NS
	Mandibular	Central incisor	6.25	5.32	7.18	6.32	5.43	7.21	6.16	5.18	7.15	1.471	.263	NS
		Lateral incisor	7.28	6.44	8.11	7.36	6.48	8.24	7.20	6.45	7.96	1.808	.028	Sig
		Canine	9.26	8.13	10.40	9.34	8.19	10.49	9.19	8.09	10.30	1.483.	.139	NS
		First premolar	9.86	8.80	10.92	10.00	8.99	11.00	9.72	8.66	10.79	2.156	.005	Sig
		Second premolar	10.83	9.37	12.29	10.97	9.36	12.58	10.68	9.43	11.94	1.781	.013	Sig
		First molar	6.42	5.62	7.22	6.58	5.88	7.28	6.20	5.31	7.09	3.368	.001	Sig
		Second molar	12.05	10.47	13.64	12.26	10.74	13.77	11.84	10.27	13.42	2.213	.001	Sig

For testing the differences in median time of emergence between boys and girls, we added binary independent variables (1 = girl, 0 = boy), so the equation is: ln(P/(1-P)) = b_0_ + b_1_AGE + b_2_GIRL. If there is a difference between the genders, the parameter for this variable is statistically significant and vice versa. The advantage of this simple approach is apparent in the possibility of expressing the likelihood (exponential value of parameter b_2_) of girls to have an individual tooth, in comparison with boys of the same age (age is controlled in the model). As expected, there was a statistically significant difference between boys and girls in most cases. The probability of girls having an individual tooth is two times higher than in boys of the same age. However, there were no significant differences in the emergence times between the two genders in the following teeth: 14, 15, 17, 21, 24 25, 27, 31, 33 and 41 (Table 1).

**Fig. 2:**
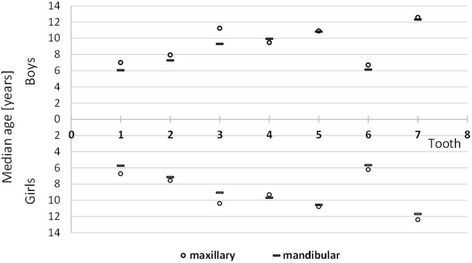
Median ages (years) of maxillary and mandibular permanent tooth emergence in Czech boys and girls (right side)

The median times of the emergence of teeth in the upper and lower jaws were tested by the McNemar test. Statistically significant inter-maxillary differences were found for all teeth with the exception of the second premolars, which were statistically insignificant. These teeth do not precede their antagonists in the lower jaw, except for the first left premolar, which is advanced in its emergence in the upper jaw (Fig. 2). With the same statistical test, the emergence times of teeth on the right and left sides were compared. For all teeth, the differences between the median ages of emergence of contra-lateral tooth pairs were not statistically significant and *P*-value was higher than 0.05. Therefore, the data are visually presented (Fig. 2) for the right side only.

The tooth eruption sequence based on the median emergence times of the right side permanent teeth was different in both genders (see Table 2). The first transitional period of the replacement of deciduous teeth begins with the emergence of the mandibular permanent central incisors in boys at the median age of 6.41 years old, and the mandibular permanent molars in girls at the median age of 6.17 years. This period is finished with the emergence of the maxillary lateral incisors in boys and girls aged 8.03 years and 7.61 years respectively. In girls, the second transitional period begins at the median age of 9.12 years, and is completed with the emergence of the maxillary second molar at the age of 12.48 years. Within the dental arch, the sequence of tooth eruption in the upper jaw commences with the first premolar, followed by the canine, second premolar and finally, the second molar. On the contrary, second premolars emerge before canines in the upper jaw in boys. The tooth emergence sequence in the lower jaw is the same for both genders: canine, first premolar, second premolar and second molar. In boys, during the second phase, the first emerged tooth is the mandibular canine at the age of 9,41 years, and the last emerged tooth (excluding the third molar) is maxillary second molar at the age of 12.67 years. The lag phase between the first and second transitional periods lasts 1.51 years in girls and 1.38 years in boys.

**Table 2:** The sequence of permanent teeth emergence for boys and girls

Boys				Girls			
Tooth	Median	Percentile		Tooth	Median	Percentile	
		5th	95th			5th	95th
Mandibular central incisor	6.41	5.57	7.25	Mandibular first molar	6.17	5.19	7.14
Mandibular first molar	6.47	5.69	7.25	Mandibular central incisor	6.21	5.24	7.18
Maxillary first molar	6.86	6.35	7.37	Maxillary first molar	6.62	5.98	7.26
Maxillary central incisor	7.04	6.72	7.36	Maxillary central incisor	6.89	6.32	7.46
Mandibular lateral incisor	7.34	6.60	8.07	Mandibular lateral incisor	7.18	6.47	7.88
Maxillary lateral incisor	8.03	6.73	9.33	Maxillary lateral incisor	7.61	6.53	8.70
Mandibular canine	9.41	8.33	10.49	Mandibular canine	9.12	8.06	10.17
Maxillary first premolar	9.55	8.40	10.69	Maxillary first premolar	9.37	8.22	10.52
Mandibular first premolar	10.01	9.00	11.02	Mandibular first premolar	9.75	8.56	10.93
Mandibular second premolar	10.89	9.49	12.29	Maxillary canine	10.45	9.19	11.71
Maxillary second premolar	10.98	9.59	12.38	Mandibular second premolar	10.62	9.40	11.84
Maxillary canine	11.32	9.98	12.67	Maxillary second premolar	10.86	9.51	12.21
Mandibular second molar	12.41	10.66	14.17	Mandibular second molar	11.81	10.16	13.45
Maxillary second molar	12.67	11.03	14.32	Maxillary second molar	12.48	11.25	13.71

## Discussion

In cross-sectional studies, the risk of bias is smaller than in longitudinal ones [29]. With respect to this, the study protocol was conceived as a cross-sectional anthropological survey focusing on emergence times and sequence of permanent teeth in Czech children, representing a Central European population sample. We can assume our study results to be uninfluenced by cases of tooth agenesis, due to the fact that the sample size was reasonably large. The incidence of dental agenesis in the Czech Republic is 7,4% (Ginzelová, 2013). In representative study samples, there is practically no systematic distortion of the results (measurement bias). In our study, we presumed that the potential overestimation of median emergence times, regardless of tooth agenesis, was less than 1%, with standard deviations ranging between 3 and 5%, rendering the effect of tooth agenesis on the study results negligible. However, due to the potential premature loss of deciduous teeth, which can be disclosed through a retrospective or longitudinal case study protocol, this might have been more appropriate [2].

Dental age can be determined by comparing the number of emerged teeth in the oral cavity of the examined child with standard values obtained by the examination of children of the same age. This simple method is used even today, although its accuracy is questionable. Tooth emergence is affected by exogenous factors and hormones of the relatively unstable thyroid gland. Thus, its correlation with bone age and chronological age is relatively low [30]. More accurate methods to determine dental age are based on the assessment of mineralization of hard dental tissues, as tooth development is controlled by the relatively stable pituitary gland [31]. In order to determine dental age based on mineralization and the stage of root development, it would be necessary to conduct a study based on a radiological examination of the subjects.

It was observed that in girls, teeth 11, 12, 13, 16, 42, 43, 44, 45, 46, 47, 22, 23, 26, 32, 34, 35, 36, 37 erupted earlier than in boys, which is attributable to earlier sexual maturation. In the Czech population sample, the greatest difference in emergence times between the genders was observed in the upper canines. Similar conclusions have been made by numerous research projects in other countries [2, 32, 5, 33–39]. As opposed to these investigations, Khan [25] found no differences between the genders in the emergence times of permanent teeth (except for teeth 15, 25 and 43). His conclusions are consistent with those from the Russian [40] and German [41] surveys. In the Czech paediatric population, no statistically significant difference was found in emergence times between the right and left side of the jaw. Similar findings were reported by authors from other countries investigating different populations [2, 33, 37–46, 34, 47, 25, 36]. In our study, the precedence in emergence of mandibular teeth (with exception of first and second premolars) is in agreement with what was reported by other researchers [2, 4, 33, 42, 48, 43, 49, 38, 32].

Therefore, for valid clinical assessment of the timing and sequence of tooth emergence in a child, it is always recommended that dental practitioners refer to the standards of tooth emergence derived from the population to which that child belongs.

The sequence of emergence of the permanent teeth is linked to ethnic [6], gender [33, 42] as well as environmental factors [13, 43, 50, 51, 34]. Therefore, for a valid clinical assessment of the sequence of tooth emergence, it is recommended that the standards derived from the population to which they are applied, be used. The simplest approach for constructing a sequence of permanent teeth emergence is based upon putting the medians of tooth emergence ages in an ascending order, as carried out in many previous studies to which we are comparing our results [49, 2, 47, 52, 53, 37]. In our male sample study, it was found that the first tooth to emerge in the oral cavity was the permanent lower central incisor. This “incisor” pattern was not only observed in our study sample, but also in Lithuanian [2], Belgian [54] and Finnish research [8]. Other studies observed that the first tooth to emerge in the oral cavity was the first permanent lower molar [49, 32, 53, 52]. This “molar” pattern was observed for Czech girls as well. The emergence sequence during the second transitional period observed in this study for both genders is comparable to similar studies from other countries [32, 53, 49, 47]. The results of our study show that the sequence of tooth emergence in the upper jaw differs according to gender, which is confirmed in other studies [53, 2]. In girls, the sequence is as follows: first premolar, canine, second premolar and second molar, a pattern also seen in other studies [49, 3]. On the other hand, in boys, second premolars emerge before canines, which was also observed previously [2]. This order is considered unfavourable in terms of possible dental crowding in the upper jaw, because the emergence of premolars before canines tends to create a shortage of space in the dental arch for the canines. The tooth emergence sequence in the mandible is the same for both genders, which is as follows: canine, first premolar, second premolar and second molar, and this sequence is in accordance with other studies [53, 3, 2]. This approach for presenting of emergence sequence of permanent teeth might be considered insufficient for the prediction of the tooth emergence sequence in an individual child because the frequencies of sequence variability have not been taken into consideration [54]. Polymorphic variations in the sequence of tooth emergence of Czech children will be the aim of future research.

On the basis of available foreign studies, it was possible to compare our data in both genders (on the right side of the upper and lower jaws) with similar research studies that were carried out in recent years in Belgium, Finland, Lithuania, Germany, Spain, the United Kingdom, Croatia, Turkey, Jordan, Iran, Ghana, Nigeria, the United States and Australia (see Tables 3 and 4). It can be seen that the median ages for teeth emergence in Czech children are more similar to European countries and others of Caucasians origin (such as Australians and North Americans) probably due to genetic affinity, than those of Asia or Africa, which are made up of different diverse ethnic groups. For example, the emergence time in Czech children was found to be significantly different from those in Ghana [55], whose teeth emerge much earlier.

**Table 3:** Overview of studies with median emergence age of permanent teeth in girls

Continent				Europe					Asia			Africa	America	Australia
Country		CZE	BEL	FIN	LTU	DEU	ESP	GBR	TUR	JOR	IRN	GHA	USA, Oregon	AUS
Year of publication		Current study	2003	1999	2012	2006	2008	2000	2004	2012	2004	1967	1978	2003
Maxilla	Central incisor	6.9	6.9	6.8	6.8	6.7	6.9	7.2	7.3	7.1	7.6	6	7.2	7.2
	Lateral incisor	7.6	7.9	7.6	7.6	7.5	7.4	8.2	8	8.1	8.8	7.3	8.2	8.2
	Canine	10.4	11.0	10.8	10.5	10.9	11.0	11.4	10.5	11.1	12.1	9.5	11	11.2
	First premolar	9.4	10.4	10.3	9.5	10.1	10.4	10.9	10.3	10.0	11.0	9.0	10.5	10.8
	Second premolar	10.9	11.4	11.6	10.6	11.1	11.2	11.8	11	11	12.5	10.0	12.2	11.7
	First molar	6.6	6.2	6.1	6.3	6.1	6.2	6.5	6.2	6.2	6.7	5.0	6.4	6.5
	Second molar	12.5	12.0	11.9	12.1	11.9	12.2	12.4	12.3	12.3	12.5	10.9	12.1	12.3
Mandible	Central incisor	6.2	6.2	5.9	5.9	6.3	6	6.4	6.7	6.3	6.5	5.1	6.1	6.3
	Lateral incisor	7.2	7.1	6.8	6.9	6.8	7.3	7.4	7.6	7.3	7.9	6.3	7.3	7.4
	Canine	9.1	9.7	9.7	9.6	9.5	9.8	10.3	10	9.8	10.3	8.9	9.9	10.1
	First premolar	9.7	10.3	10.3	9.7	10.1	10.3	10.7	10.2	10.1	11.1	9.2	10.4	10.6
	Second premolar	10.6	11.4	11.3	10.6	10.9	11.3	11.9	11.1	11.2	12.6	10.3	11.1	11.7
	First molar	6.2	6.2	6.1	6.0	6.1	6.1	6.5	6.2	6.1	6.7	4.4	6.3	6.3
	Second molar	11.8	11.6	11.6	11.3	11.2	11.5	12	11.9	11.7	12.4	10.5	11.8	11.8

*Abbreviations*: *CZE* Czech Republic, *BEL* Belgium (Leroy et al., 2003 [43]), *FIN* Finland (Eskeli et al., 1999 [42]), *LTU* Lithuania (Almonaitiene et al., 2012 [2]), *DEU* Germany (Friedrich et al., 2006 [38]), *ESP* Spain (Hernandez et al., 2008 [48]), *GBR* United Kingdom (Elmes et al., 2010), *TUR* Turkey (Wedl et al., 2004a [35]), *JOR* Jordan (Shaweesh, 2012) [56], *IRN* Iran (Moslemi, 2004 [47]), *GHA* Ghana (Houpt et al., 1967) [55], *USA* the United States of America (Savara & Steen, 1978 [57]), *AUS* Australia (Diamanti & Townsend, 2003 [33]

**Table 4:** Overview of studies with the median emergence age of permanent teeth in boys

Continent		Europe							Asia			Africa	America	Australia
Country		CZE	BEL	FIN	LTU	DEU	ESP	GBR	TUR	JOR	IRN	GHA	USA, Oregon	AUS
Year of publication		Current study	2003	1999	2012	2006	2008	2000	2004	2012	2004	1967	1978	2003
Maxilla	Central incisor	7.0	7.1	6.8	6.9	6.7	7.2	7.4	7.1	7.3	6.8	6.2	7.2	7.4
	Lateral incisor	8.0	8.3	8.1	8.0	8.0	8.2	8.8	7.9	8.5	8.4	7.4	8.3	8.6
	Canine	11.3	11.5	11.3	11.1	11.2	11.6	12	10.8	11.6	11.8	10.0	11.5	11.8
	First premolar	9.5	10.7	10.9	9.9	10.5	10.9	10	10.2	10.5	12.0	9.3	11.1	11.3
	Second premolar	11.0	11.6	11.7	10.8	11.4	11.5	12.3	11	11.4	12.0	10.3	11.7	12.1
	First molar	6.9	6.3	6.3	6.4	6.2	6.3	6.8	5.9	6.4	6.8	5	6.5	6.7
	Second molar	12.7	12.3	12.4	12.3	12.5	12.5	12.8	12.2	12.6	12.7	10.9	12.2	12.7
Mandible	Central incisor	6.4	6.3	6.0	6.1	6.3	6.3	6.6	6.6	6.5	6.0	5.2	6.2	6.6
	Lateral incisor	7.3	7.4	7.1	7.2	7.1	7.5	7.8	7.6	7.5	7.3	6.3	7.5	7.8
	Canine	9.4	10.6	10.5	10.4	10.5	10.6	11	10.2	10.6	9.7	9.5	10.7	11.0
	First premolar	10.0	10.7	10.7	10.1	10.5	10.7	11.2	10.2	10.5	10.1	9.5	10.9	11.2
	Second premolar	10.9	11.7	11.6	11.1	11.4	11.7	12.2	11	11.7	10.9	10.5	11.6	12.1
	First molar	6.5	6.3	6.2	6.2	6.1	6.3	6.8	6.0	6.2	5.6	4.7	6.5	6.6
	Second molar	12.4	11.8	12.0	11.7	11.7	12.0	12.3	11.9	12.2	11.3	10.6	12.0	12.2

*Abbreviations*: *CZE* Czech Republic, *BEL* Belgium (Leroy et al., 2003 [43]), *FIN* Finland (Eskeli et al., 1999 [42]), *LTU* Lithuania (Almonaitiene et al., 2012 [2]), *DEU* Germany (Friedrich et al., 2006 [38]), *ESP* Spain (Hernandez et al., 2008 [48]), *GBR* United Kingdom (Elmes et al., 2010), *TUR* Turkey (Wedl et al., 2004a [35]), *JOR* Jordan (Shaweesh, 2012) [56], *IRN* Iran (Moslemi, 2004 [47]), *GHA* Ghana (Houpt et al., 1967) [55], *USA* the United States of America (Savara & Steen, 1978 [57]), *AUS* Australia (Diamanti & Townsend, 2003 [33])

## Conclusion

Our study document reference values for permanent teeth emergence in Czech children. The girls emerged their teeth earlier than the boys. The greatest difference in the timing of emergence was observed in maxillary canines. The sequence of emergence among Czech children does not differ from published results from other populations.
